# Illuminating dynamic neutrophil trans-epithelial migration with micro-optical coherence tomography

**DOI:** 10.1038/srep45789

**Published:** 2017-04-03

**Authors:** Kengyeh K. Chu, Mark E. Kusek, Linbo Liu, Avira Som, Lael M. Yonker, Huimin Leung, Dongyao Cui, Jinhyeob Ryu, Alexander D. Eaton, Guillermo J. Tearney, Bryan P. Hurley

**Affiliations:** 1 Department of Dermatology, Harvard Medical School, Boston, MA, USA; 2 Department of Pediatrics, Harvard Medical School, Boston, MA, USA; 3 Wellman Center for Photomedicine, Boston, MA, USA; 4 Mucosal Immunology and Biology Research Center, Boston, MA, USA; 5 Department of Pathology, Massachusetts General Hospital, Boston, MA, USA

## Abstract

A model of neutrophil migration across epithelia is desirable to interrogate the underlying mechanisms of neutrophilic breach of mucosal barriers. A co-culture system consisting of a polarized mucosal epithelium and human neutrophils can provide a versatile model of trans-epithelial migration *in vitro*, but observations are typically limited to quantification of migrated neutrophils by myeloperoxidase correlation, a destructive assay that precludes direct longitudinal study. Our laboratory has recently developed a new isotropic 1-μm resolution optical imaging technique termed micro-optical coherence tomography (μOCT) that enables 4D (x,y,z,t) visualization of neutrophils in the co-culture environment. By applying μOCT to the trans-epithelial migration model, we can robustly monitor the spatial distribution as well as the quantity of neutrophils chemotactically crossing the epithelial boundary over time. Here, we demonstrate the imaging and quantitative migration results of our system as applied to neutrophils migrating across intestinal epithelia in response to a chemoattractant. We also demonstrate that perturbation of a key molecular event known to be critical for effective neutrophil trans-epithelial migration (CD18 engagement) substantially impacts this process both qualitatively and quantitatively.

Mucosal surfaces operate as a barrier comprised of tightly connected epithelial cells adapted in an organ-specific manner for extracting beneficial resources while excluding harmful threats. Epithelial-orchestrated innate immunity can help deploy defense measures such as rapid mobilization of circulating neutrophils. Recruitment of neutrophils (PMNs) from the bloodstream to the mucosal lumen is a highly complex process involving multiple cytokines, chemo-attractants, adhesion molecules, and other mediators, each acting at discrete compartments to direct PMNs across injured or infected mucosal tissue^1^,^2^,^3^,^4^,^5^. PMNs attach at distress sites on activated endothelial cells in proximity to injured tissue, then must navigate endothelial barriers, interstitial space, and the mucosal epithelial barrier^1^,^2^,^3^,^4^,^5^. Following migration, PMNs then either remain tethered to the apical surface to perform antimicrobial activities or detach from the epithelium and enter the lumen^1^,^2^,^3^,^4^,^5^.

Abnormal neutrophil migration contributes to several pathologies. Inflammatory bowel disease is a chronic inflammatory condition that features an inappropriate and unexplained pathological PMN penetration of the digestive tract mucosal barrier^1^,^6^. Additional infectious and auto-inflammatory conditions also present with excessive neutrophil trans-epithelial migration at mucosal surfaces, suggesting that neutrophilic breach of the mucosal barrier is likely a significant pathological event contributing to a variety of disease processes.

Trafficking of immune cells through tissue compartments is difficult to study in context^4^,^5^,^7^. *In vivo* models provide evidence for the role of key molecular players such as adhesion molecules and chemotactic agents^8^,^9^, but are complicated by the confounding factors in the molecular and cellular mechanisms that define migration through each discrete tissue compartment. *In vitro* co-culture systems are used widely to disentangle the mechanisms of PMN trans-epithelial migration^4^,^7^,^10^ Mucosal epithelial cell lines generate polarized functional barriers when grown on permeable Transwell filters^2^,^4^,^7^,^11^,^12^,^13^,^14^,^15^, and can be cultured on the underside of permeable Transwells with the apical side facing down. This orientation allows for the addition of PMNs to the basolateral side and examination of migration in the physiologically correct direction by establishing a chemo-attractant gradient on the apical side or treating the epithelium with cytokines or pathogens to promote polarized chemotactic gradient formation 4,7,10,12. Several key molecular adhesion events occurring between PMNs and epithelial cells as well as chemotactic drivers have been revealed by *in vitro* co-culture modeling with their importance to recruitment confirmed *in vivo* through carefully designed experiments in mice^2^,^4^,^7^,^8^,^9^. Molecular mechanisms of PMN trans-epithelial migration have been well characterized using the polarized T84 intestinal epithelial cell line grown inverted on permeable Transwell filters^2^,^16^.

Various forms of microscopic imaging have been utilized to observe immune cells in action to gain a better understanding of cellular mechanisms that define their behavior. Fluorescently labeled PMNs are amenable to standard fluorescence imaging techniques, including widefield and confocal^17^,^18^, though such methods generally constrain experimentation to transgenic animal-derived cells and are of lower resolution in the axial direction. Exogenous fluorescent labeling also risks altering the migratory phenotype. Optical coherence tomography (OCT) is a cross-sectional depth ranging imaging technology that interferometrically senses back-scattered and reflected light, forming depth-resolved images that do not require exogenous contrast. Though the ability to image unlabeled cells in 3D is ideally suited for immune cell imaging, the typical resolution performance of conventional OCT is insufficient to resolve individual immune cells of approximately ~10 μm diameter.

More recently, our laboratory has developed micro-OCT (μOCT)^19^, which is an implementation of OCT with a one-micron axial resolution. μOCT has been successfully applied to the air-liquid interface model of bronchial epithelium where the high resolution and native contrast provides a clear visual resolution of beating cilia and mucus propulsion^20^. Attainment of more detailed visual information has facilitated the development of useful quantitative metrics including measurement of airway surface liquid depth, cilia beat frequency, and mucociliary transport rate all within a single sample^20^. Such technology pairs image acquisition with multiplexed quantification of previously inaccessible micro-physiological processes of live mucosa.

In this study, we have applied μOCT to gain a deeper understanding of micro-anatomical dynamics of neutrophilic mucosal barrier breach. Because individual PMNs are resolvable by μOCT, intercellular interaction can be directly observed. We present high-resolution images and videos of intestinal epithelial monolayers and neutrophils during and after neutrophil migration through inverted epithelial cultures. We also illustrate the application of quantitative assays to μOCT images, allowing PMN migration to be quantified longitudinally over time without perturbing the trans-epithelial migration model and without introducing error due to sample variability that is inevitable with any endpoint destructive assay. Finally, we utilized the μOCT imaging and quantitative analysis methods to explore the consequences of interference with CD18 adhesion receptor. CD18 is critical for neutrophil trafficking through various compartments including the epithelial barrier^16^. The importance of CD18 is underscored by the severe immuno-deficiency and recurrent infections experienced by patients that have Leukocyte Adhesion Deficiency I, a condition defined by a mutation in the gene that encodes CD18^21^.

## Materials and Methods

### Culturing epithelial cells

Colorectal carcinoma human intestinal epithelial cell line T84 was purchased from American Tissue Culture Collection (ATCC, Manassas, VA). T84 cells were cultured in DMEM/F12 (Invitrogen, Carlsbad, CA) supplemented with 10% heat inactivated fetal bovine serum and 1X Penicillin-Streptomycin (Invitrogen, Carlsbad, CA). To obtain polarized epithelial monolayers, T84 epithelial cells were cultured on 0.33 cm^2^ growth area collagen coated permeable Transwell filters (Corning Incorporated, Corning, NY) as previously described^7^,^12^,^13^,^14^,^15^. For experiments involving imaging and assessment of the T84 barrier, cells were grown in the conventional manner by seeding cells within 0.4 μm pore sized Transwells and allowing at least one week for growth and formation of polarized monolayers. For neutrophil migration assays, 3.0 μm pore sized Transwell filters were inverted and epithelial cells were seeded on underside and incubated overnight. The following day, Transwells were flipped back into a 24-well plate, bathed on both sides with media, and incubated for at least one week to allow growth and barrier formation.

### Assessment of T84 barrier properties

Measurement of trans-epithelial electrical resistance (TEER) and flux of ^3^H-inulin from the apical to basolateral compartment of T84 polarized monolayers cultured on Transwells was conducted in the presence and absence of 5 mM EDTA for 24 hours. TEER was measured as Ω/monolayer using the EVOM2, Epithelial Voltohmmeter (World Precision Instruments, Inc., Sarasota, FL). For ^3^H-inulin flux, 2.5 μCi/ml ^3^H-inulin (PerkinElmer Inc. - NEN [New England Nuclear], Wellesley, Massachusetts, USA) was added to the apical surface and the amount of ^3^H-inulin that reached the basolateral compartment after 24 hours in the presence or absence of EDTA was determined by sampling counts per minute (cpm) using an LS 6500 Multi-purpose Scintillation Counter from Beckman Coulter, Inc. (Brea, CA). Data is presented as % flux of ^3^H-inulin where the amount measured in the basolateral compartment adjusted for volume is divided by the total amount added to the apical surface and multiplied by 100^22^.

### Neutrophil isolation

Neutrophils were isolated from the blood of healthy volunteers and informed consent was obtained from each participant in accordance with human studies protocol #1999P007782 approved by the Institutional Review Board of Massachusetts General Hospital. A complete description of the neutrophil isolation protocol was previously published^10^. Briefly, whole blood was subjected to centrifugation to remove plasma and mononuclear cells followed by sedimentation of RBCs using 2% gelatin. To remove residual RBCs, cells were treated with cold ammonium chloride lysis buffer leaving the granulocyte cell fraction intact, which consists of greater than 90% neutrophils^23^. Neutrophils were resuspended in Hank’s balanced salt solution without calcium or magnesium (HBSS-) at a concentration of 5 × 10^7^ cells/ml and placed on ice until initiation of experiment^10^,^12^.

### Neutrophil trans-epithelial migration

To initiate neutrophil trans-epithelial migration, 1 × 10^6^ neutrophils were placed in the basolateral well of the inverted T84 intestinal epithelial monolayers grown on the underside of the Transwell filter. At the same time, a gradient of neutrophil chemo-attractant was established by placing 100 nM formyl-methionyl-leucyl-phenylalanine (fMLP, also commonly abbreviated fMLF) in the apical compartment to instigate basolateral to apical neutrophil migration. Alternatively, HBSS + only was applied to the apical compartment as a negative control. Migration was allowed to proceed at room temperature or at 37 °C for various amounts of times depending on the particular experiment being conducted^12^.

### Imaging using micro-optical coherence tomography (μOCT)

Micro-optical coherence tomography (μOCT) is a 1-μm axial resolution implementation of OCT and has been previously utilized to image airway cilia and coronary arteries^19^,^20^. A broadband laser beam was focused into a sample, and the depths of reflected and scattered light are reconstructed through spectral-domain interferometry to form a 1-dimensional depth profile (A-line). Scanning mirrors directed the beam through a range of lateral positions to form 2D frames or 3D volume images. The high axial resolution of μOCT was derived from the extremely broadband 650–950 nm laser illumination provided by the NKT Photonics (Birkerod, Denmark) SuperK Extreme OCT supercontinuum laser. High lateral resolution (2 μm) and long depth of focus were achieved by annular apodization as previously described^19^. The interferometer optics were mounted on a rotatable vertical board to allow imaging from above or below a sample.

Neutrophil migration on inverted Transwells was imaged by μOCT in the inverted configuration. A customized sample holder was constructed to hold Transwells in an open chamber with a transparent bottom and a 100 μm silicone spacer to standardize the distance between the epithelial monolayer and the glass chamber bottom. Imaging and reference power were standardized for each experiment to allow consistent intensity-based quantitative analysis (see below). The imaging platform was rotated approximately 8 degrees relative to the μOCT imaging beam to minimize direct reflections from flat surfaces such as the glass bottom or the Transwell filter. For migration studies, a 75-watt incandescent light bulb was illuminated approximately 3 inches from the sample and an aluminum foil duct was placed to the transfer heat from the bulb to the sample. The outside of the chamber walls was coated with black enamel to increase radiative absorption, and the position of the foil duct was adjusted to produce a sample temperature of approximately 37 °C. Some migration experiments were performed at room temperature, prior to the installation of the heating lamp. These experiments are so labeled in the results.

2D images were acquired by scanning the OCT beam in a linear path (B-scan) over a 1 mm length region. The 3D images are created by acquiring multiple B-scans by sweeping a 1mm x 1mm area (12.8 seconds). Custom acquisition software was modified to allow programmable time-lapse operation; 13 images taken at 10 minute intervals were typically acquired for a two-hour length experiment. Typical Fourier-domain OCT reconstruction was applied to convert raw interferometric data to depth-resolved images^24^, which were rendered as 3D volumes or as cross-sections for viewing and analysis.

### Quantitative analysis of neutrophil trans-epithelial migration using myeloperoxidase activity

Upon setting up a neutrophil trans-epithelial migration experiment as described above, once neutrophils were added to the basolateral surface (t = 0), migration was allowed to proceed for various time points (30 min., 60 min., 90 min., and 120 min.) until terminated by removing the Transwell at the designated time. Upon removal, Transwells were washed at least three times in HBSS + to remove any loosely associated neutrophils. Both Transwells containing monolayer associated neutrophils in the process of migrating and apical compartments containing fully migrated neutrophils were treated with 0.5% triton-X-100 and 50 mM citrate. The number of neutrophils in each sample was quantified using myeloperoxidase (MPO) activity. Briefly, peroxidase activity in disrupted cells was analyzed by addition of 2,2′-Azino-bis(3-ethylbenzothiazoline-6-sulfonic acid (ABTS) and H_2_O_2_ (Sigma-Aldrich, St. Louis, MO). The optical density at 405 nm was then measured and converted to a neutrophil count by referencing a previously derived calibration curve relating optical density to a range of neutrophil numbers obtained through serial dilution (approximately 1 OD increase per 5 × 10^4^ neutrophils)^10^. Background peroxidase activity associated with epithelial cells was quantified from parallel monolayers in the absence of neutrophils and subtracted from experiments containing neutrophils.

### Quantitative analysis of neutrophil trans-epithelial migration using μOCT

Experiments involving neutrophil migration across inverted T84 monolayers grown on Transwells that were analyzed by μOCT were prepared identically to experiments analyzed using the MPO assay, with the exception that longitudinal experiments were made possible by μOCT analysis with multiple time points measured from the same region of the same samples. μOCT 3D time-lapse imaging was performed as described above. Image manipulation software (ImageJ) was used to load and view 3D datasets from each time point into 8-bit “hyperstacks,” a 4D ImageJ format. The anti-reflection 8° tilt was removed by rotation with bilinear interpolation. A threshold was then applied to the hyperstack such that voxels above a given limit were assigned maximum intensity (255), and those below were assigned minimum intensity (0). A projection was conducted to collapse the hyperstack by summing all pixels through the third dimension, reducing the data to a 2D image for each of the 13 time points. A region of interest was drawn around the location of the glass chamber surface and adjacent to the epithelial surface, and the sum of bright pixels located therein for each time point was measured and recorded. We separately counted neutrophils in regions adjacent to the epithelium (<60 μm distance) and those more distant; these cells were respectively classified as “attached” or “detached” from the epithelium, because unadhered neutrophils settle to the chamber bottom after migration under the influence of gravity. The value obtained at t = 0 was subtracted from all subsequent time points to normalize for the unchanging reflectivity of the surfaces themselves; no neutrophils were presumed to be present in this first measurement.

The 3D μOCT volumes were acquired at 10 minute intervals, chosen for a balance of temporal resolution and data economy. The 3D volumes were acquired in 12.8 seconds each; the time lapses could be thus theoretically acquired at approximately 4 volumes per minute. We performed quantification solely on 3D images; while the same methods would be applicable to 2D cross-sections, the 3D datasets sampled a much larger region of the epithelium and were thus more appropriate for statistical comparisons.

We also computed average migration distance of neutrophils crossing an epithelial barrier for some datasets. The thresholded hyperstacks from the above analysis were imported into MATLAB (Mathworks, Natick, MA). The percentage of above-threshold pixels on each horizontal plane was computed and assigned to the vertical distance separating the plane and the Transwell filter, generating a curve of neutrophil occupation percentage as a function of distance for each time point of each experiment. The centroid of each distribution was computed, which yielded the average migration distance of neutrophils.

### PMN Migration across T84 with anti-CD18

An optimal concentration of antibody for pre-treatment of neutrophils to achieve partial reduction in trans-epithelial migration was determined by pre-treating neutrophils with antibody concentrations between (1 and 10 μg/ml) of either isotype control antibody (IgG1) or anti-CD18 antibody for 30 minutes on ice prior to adding neutrophils to the basolateral side of the epithelium. The numbers of neutrophils that fully migrated across T84 monolayers in response to fMLP were quantified by MPO activity. Anti-CD18 [MEM-48] antibody, low endotoxin, azide free and mouse IgG1 monoclonal (NCG01) isotype control were purchased from Abcam (Cambridge, MA).

Using this antibody concentration, we performed a series of controlled experiments, comparing neutrophils treated with anti-CD18 with those treated with IgG1 isotype control. We performed 6 iterations of both conditions using μOCT 3D time lapse, each treatment experiment paired with a same-day control to account for any variability in neutrophils or T84 epithelial cultures. Following isolation, neutrophils were treated with 5 μg/ml of either anti-CD18 antibody or IgG1 isotype control for 30 minutes on ice. After anti-CD18 treatment or isotype control application, the neutrophils were placed in the basolateral compartment as before. μOCT volumes of 1 mm × 1 mm × 400 μm were acquired every 10 minutes for 2 hours. Experiments were conducted on six separate days, with each anti-CD18 paired with a same-day isotype control application of IgG1. Because the μOCT imaging was performed sequentially on control and anti-CD18 treatment, the experiment order was alternated to eliminate any possible timing effect. Three pairs were imaged control-first, and the remaining three pairs were imaged anti-CD18 first.

### Statistical analysis

Quantitative data were plotted and analyzed using Prism 7.0 (GraphPad, La Jolla, CA). Sample means were presented in figures with error bars representing standard error of the mean (SEM).

To compare anti-CD18 and IgG1 isotype control treated neutrophil results, statistical comparisons were made between the neutrophil counts, adhesion ratios, and migration distances of the corresponding time points. Two-sample t-tests were performed between the metrics at each time point to test the null hypothesis that the tested metric was equal between the treatment and control (n = 6 for each group, df = 10). A p-value of less than 0.05 was considered significant.

## Results

### T84 monolayer: cross-section and 3D rendering by μOCT

**Figure 1:**
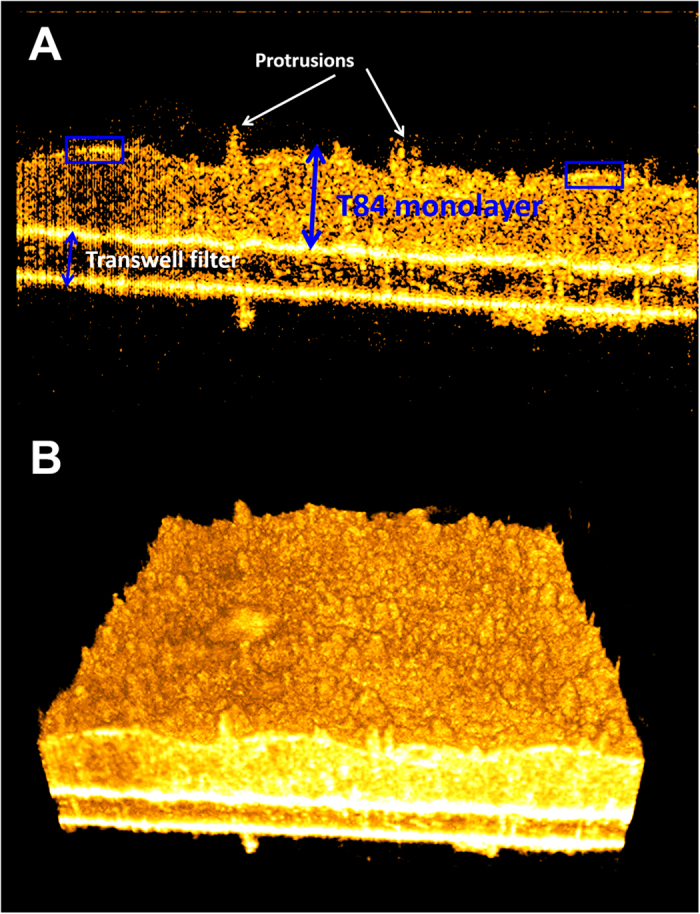
μOCT images of T84 epithelium. (**A**) XZ cross-sections. The monolayer (larger blue double sided arrow) is cultured upon a porous Transwell filter (smaller blue double sided arrow). Notable features include apical protrusions (white arrows) and brightly reflective apical surface (blue boxes). (**B**) 3D rendering showing apical epithelium.

A μOCT image of intestinal epithelial T84 cells grown on the Transwell reveals a continuous monolayer closely associated with the collagen coated permeable filter (Fig. 1). The μOCT image represents a cross-sectional XZ perspective; the horizontal X axis is parallel to the Transwell surface, while the vertical Z axis represents depth. The permeable filter is observed as two bright lines with a darker area in between (Fig. 1). The apical surface, facing away from the filter, appears to be brighter than the remainder of the cell and there also appears to be a smattering of projections emanating from the apical surface (Fig. 1A).The XZ field of view as seen in Fig. 1A, when scanned in the direction perpendicular to the frame (Y), yields a 3D volume (XYZ) (Video 1, Fig. 1B).

### The Impact of barrier integrity perturbation on the T84 monolayer

**Figure 2:**
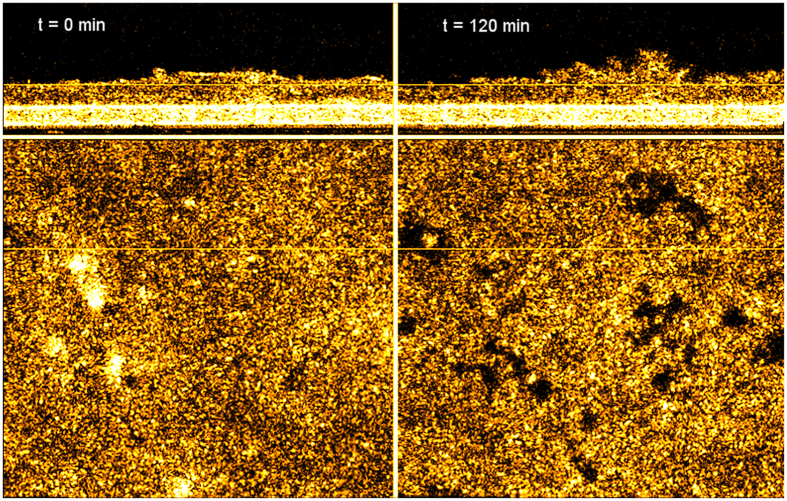
μOCT cross-sectional XZ (top) and en-face XY (bottom) views of T84 epithelium immediately prior to (left) and 2 hours after the application of EDTA (right).

Ethelenediaminetetraacetic acid (EDTA) is an aminopolycarboxylic acid which is useful as a hexadentate ligand and chelating agent. When it is applied to epithelial cells, it disrupts the tight junctions by sequestering metal ions including calcium. Exposure to EDTA impacts T84 epithelial barrier structure and function, which is visually assessable using μOCT imaging. The XZ cross-sectional image (Fig. 2, Video 2 and 3) reveals disruption and disorganization in the continuous T84 monolayer highlighted by loss of apical surface brightness and increased deviation in monolayer thickness. These visual cues are accompanied by physiological evidence of diminished barrier integrity. T84 monolayers develop transepithelial electrical resistance (TEER), which reflects barrier integrity. TEER decreases significantly when EDTA is applied to the epithelial surface (Supplementary Figure 1A). This disruption of the epithelium barrier integrity is also demonstrable through the observation of significantly increased flux of the paracellular probes 3H-inulin following T84 exposure to EDTA (Supplementary Figure 1B). Taken together, these data demonstrate that EDTA treatment elicits rapid observable changes in epithelial monolayer structure captured by μOCT that manifest functionally as loss in barrier integrity by multiple assays.

### PMN Migration across T84 monolayers in Response to fMLP Chemotactic Gradient

**Figure 3:**
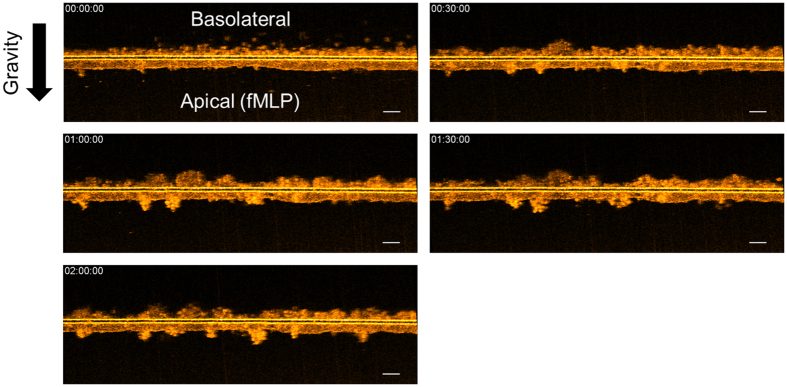
Time-lapse of μOCT cross-sectional XZ images of neutrophils traversing a T84 monolayer in response to fMLP chemoattractant added to the apical compartment. In the initial frame, neutrophils placed in the basolateral compartment are settling with gravity towards the Transwell membrane. At 30 minutes, neutrophils can be seen entering the epithelium and beginning to protrude into the apical medium. Thereafter, clusters of neutrophils can be seen protruding from the membrane. Scale bars: 50 μm. Full movie data available as Video 4.

By seeding T84 cells on the underside of 3.0 μm Transwell filters and application of PMNs to the inner well of the Transwell representing the basolateral side, trans-epithelial migration of PMNs in a physiologically relevant basolateral to apical direction can be achieved following the establishment of a chemotactic gradient on the apical side. Figure 3 shows μOCT cross-sectional views of neutrophils above the filter migrating down through the T84 epithelial monolayer in response to fMLP, a well-known PMN chemo-attractant, over a period of 2 hours, which is also presented in Video 4. On the video, neutrophils can be seen migrating in several large groups down through the Transwell, seemingly to follow each other once the barrier is breached at the distinct areas. Even after the neutrophils traversed the epithelial boundary, they remained adhered to each other and to the epithelium, constantly in motion during this process. In control experiments without fMLP, negligible migration was observed (Supplementary Figure 2).

**Figure 4:**
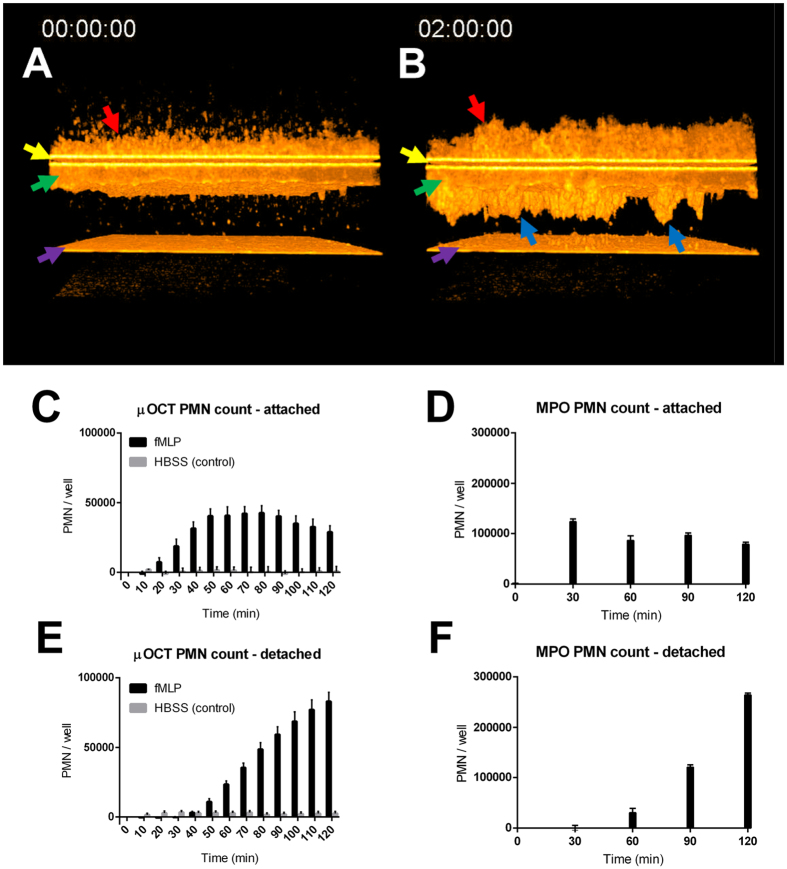
Results from μOCT time lapse of neutrophil migration through T84 epithelium driven by fMLP chemoattractant. (**A**) and (**B**) Initial and final 3D renderings of 1 mm region during migration (room temperature). Neutrophils (red arrows) added to the basolateral side of the Transwell (yellow arrows) and epithelial barrier (green arrows) at time 0 penetrate the epithelium in discrete clusters (blue arrows) over a 2 hour imaging period. A fraction of the neutrophils detach from the clusters and fall to the glass surface (violet arrows). See Video 5 for a complete time lapse at 10-minute intervals. (**C**–**F**) Comparison of quantitative migration results obtained by μOCT image analysis and myeloperixodase (MPO) assay; migration across T84 monolayers in response to fMLP. μOCT results include negative control (HBSS only). Error bars in all cases represent SEM, and PMN totals were scaled to represent the area of an entire Transwell filter. (**C**) and (**D**) Number of PMNs attached to epithelium as measured by μOCT and MPO respectively. (**E**) and F) Number of PMNs detached from epithelium as measured by μOCT and MPO respectively.

The 3D time lapses presented in Fig. 4A,B and Video 5 show similar behavior to the 2D view, with the added ability to visualize the spatial distribution of the neutrophil migration clusters over a larger region of the epithelium. 3D volumes of 1 mm × 1 mm × 300 μm were acquired every 10 minutes over a 2-hour imaging session which show the discrete clusters of migration distributed over the T84 epithelial surface. The distribution of these migration sites was heterogeneously distributed; some regions of the epithelium were densely clustered with migrating neutrophils, while other epithelial regions were largely undisturbed by neutrophils. This type of 3D time lapse qualitatively reveals similar migration behavior as the 2D case, but because a larger region of the surface is sampled, quantitative migration data can also be derived.

Previous studies have demonstrated that μOCT imaging data can be captured and analyzed quantitatively^20^,^25^. To examine neutrophil trans-epithelial migration quantitatively over time, using the standard MPO activity, distinct monolayers must be used for each time point, whereas μOCT data can be captured longitudinally within a single monolayer. Figure 4 also shows the comparisons between the μOCT-derived results (Fig. 4C and E) and those obtained by the standard myeloperoxidase (MPO) assay (Fig. 4D and F) for both in the process of migrating (Fig. 4C and D) and post-migrated PMNs (Fig. 4E and F). The time-dependent trends of the μOCT-based measurements were broadly similar to the MPO-based results quantitatively, which affirms the accuracy of the μOCT assay. The μOCT measurements represent longitudinal analysis of multiple areas within a single monolayer progressing over time while MPO-based results relied on analysis of multiple monolayer experiments processed at different time points.

### Migration of PMNs pre-treated with anti-CD18 across T84 Monolayers

Certain PMN-PMN and PMN-epithelial interactions are known to be critical towards facilitating PMN trans-epithelial migration and require specific surface receptors. A notable and well-studied example is the CD18/CD11b expressed on PMNs^16^,^26^. Pre-treatment of PMNs with antibodies against CD18 decreased PMN migration across T84 monolayers in response to fMLP in a dose dependent manner as measured by MPO activity (Supplementary Figure 3). An irrelevant isotype control antibody failed to diminish PMN trans-epithelial migration (Supplementary Figure 3). For subsequent studies, we selected a pre-treatment concentration of 5 μg/ml anti-CD18 because this resulted in a statistically significant decrease in trans-epithelial migration when compared with no antibody control, yet still permitted some degree of trans-epithelial migration.

**Figure 5:**
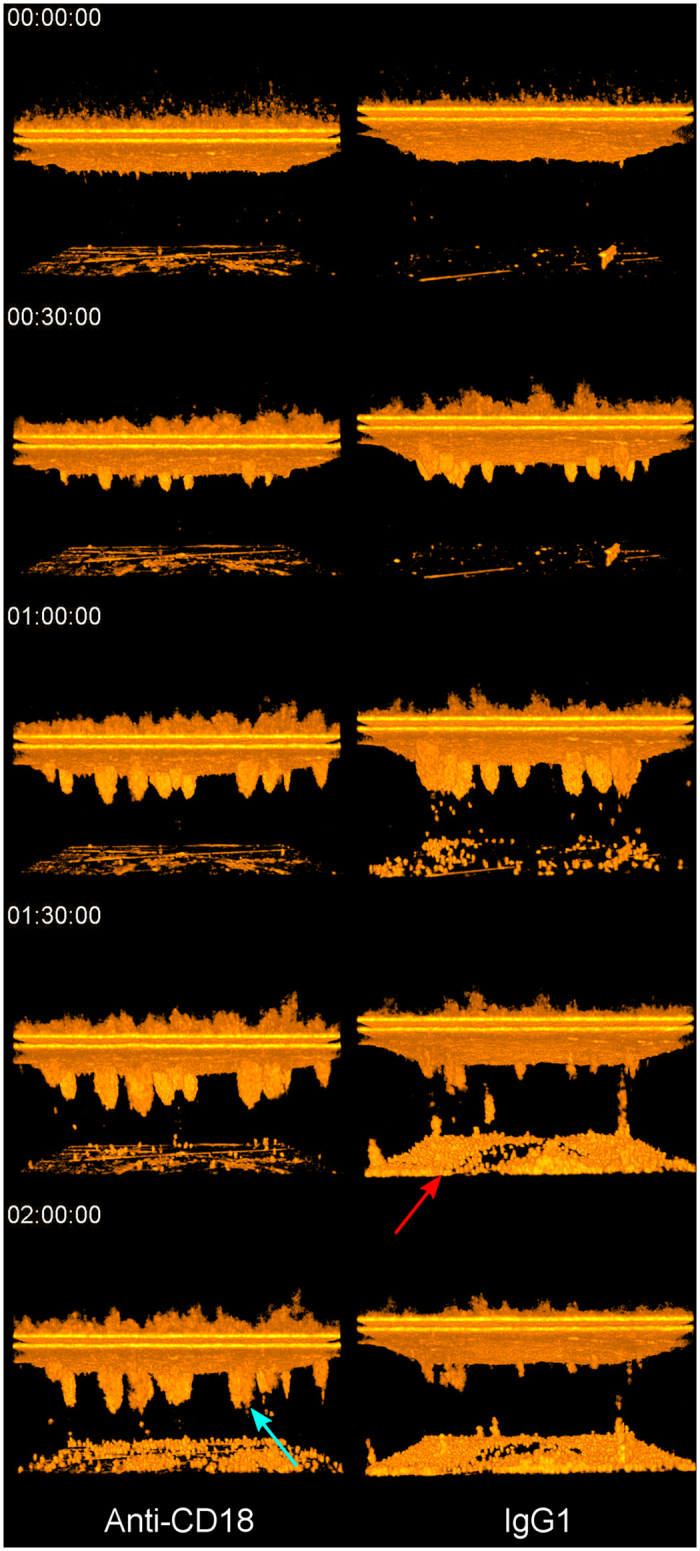
μOCT renderings of neutrophil migration through T84 in response to fMLP gradient. Time lapse through 2 hours. Left: Neutrophils pretreated with anti-CD18 antibody. Right: Neutrophils pretreated with isotype control (IgG1). IgG1-treated neutrophils detach readily after migration and aggregate at the slide chamber bottom (red arrow). Anti-CD18 treated neutrophils remain largely attached in clusters at the end of the experiment window (blue arrow).

**Figure 6:**
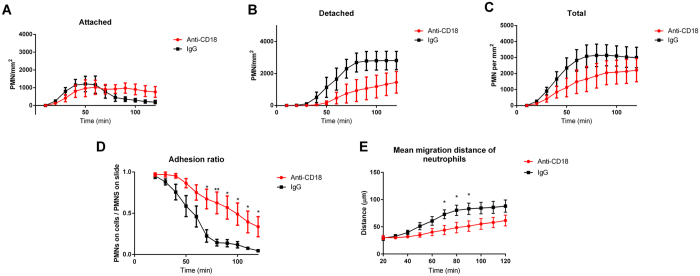
Time profile of neutrophil migration quantity across T84, measured by μOCT, anti-CD18 treated vs. IgG1 control. (**A**) Migration density of neutrophils detected within approximately 60 μm of epithelium. (**B**) Migration density of neutrophils detected not within 60 μm of epithelium. (**C**) Total migration density of neutrophils. (**D**) Ratio of neutrophils detected within 60 μm of epithelium to total neutrophils detected. E: Mean migration distance of neutrophils. All panels: Time points with significant differences between treatments denoted by *(p < 0.05) or **(p < 0.01).

The μOCT images of neutrophil migration revealed significant differences in neutrophil behavior between control and anti-CD18 treated PMNs, with a representative experiment shown in Fig. 5 and Video 6. The anti-CD18 treated neutrophils appear to migrate in clusters, which remain attached to the epithelium throughout the 2-hour experiment time. The control-treated neutrophils also migrate in clusters, but can be seen detaching individually at the 60-minute mark, and can be seen to be almost entirely detached at the end (2 hours). Results of quantitative neutrophil analysis of 6 repeated runs are shown in Fig. 6. In Fig. 6A, both treatment and control neutrophils begin their migrations with a similar magnitude and at a similar time frame, suggesting that anti-CD18 treated neutrophils were not afflicted by immobility or inability to sense the chemotactic gradient. However, neutrophils can be seen detaching after 40 minutes in the control case, and reached a plateau at approximately 70 minutes after start (Fig. 6B). The anti-CD18 treated PMNs began detaching later compared to the control, and also detached at a slower rate, never reaching equilibrium within the 2-hour experimental window.

The total migration volume, attached and detached, favored the IgG control neutrophils, though not to a statistically significant extent (Fig. 6C). The fraction of neutrophils that remained within 60 μm of the epithelium presented the starkest contrast between control and anti-CD18 (Fig. 6D). The control cases exhibited uniform detachment of post-migration neutrophils. T-tests performed between the n = 6 samples of each condition at every time point revealed statistically significant differences at t = 70 minutes and later.

As another method of quantifying epithelial adhesion differences, we also analyzed the distribution of mean distances traveled by the migrated neutrophils (Fig. 6E). The isotype control neutrophils distanced themselves quickly from the epithelium and reached a plateau near the 100 μm limit of the chamber. The anti-CD18 treated neutrophils moved more slowly, with p < 0.05 statistical significance indicated from 70 to 90 minutes, which was also the window of greatest disparity in the adhesion ratio.

## Discussion

In this study, we have shown that μOCT provides label-free volumetric images of active neutrophil migration with sufficient resolution to visualize both the neutrophils and the epithelium. The static 3D morphology of the epithelia can be imaged by μOCT as shown in Fig. 1, but the greater value from label-free non-contact imaging is tracking changes over time of the epithelium. For example, as EDTA disrupted the integrity of T84 epithelium, μOCT revealed the loss of the contiguous apical surface and the development of cellular disassociation along the epithelial monolayer and over time following exposure (Fig. 2 and Videos 2, 3). These visual observations complement measurements of trans-epithelial electrical resistance and 3H-inulin flux (Supplementary Figure 1), which both point to the loss of epithelial barrier integrity. μOCT captured morphological changes in real-time that likely led to these downstream functional effects on barrier integrity.

The ability of μOCT to acquire images over minutes or hours is quite useful for studying neutrophil behavior, as certain disadvantages of correlative endpoint MPO assays to assess the quantity of neutrophils migrating across the epithelium can be mitigated. Longitudinal imaging eliminates sample-to-sample variability that may confound analysis of time-dependent migration. Further, μOCT imaging captures aspects of neutrophil and epithelial behavior during migration that are not revealed in the context of analysis with traditional methods, as evidenced by the images of neutrophils migrating across a T84 intestinal epithelial barrier in response to an fMLP chemotactic gradient. Figures 3 and 4 and Movies S4–S5 illustrate the capabilities of both high-speed 2D imaging and time-lapsed 3D imaging of migrating neutrophils. Notably, the neutrophils migrated through the epithelium in discrete clusters.

In addition to a wealth of descriptive data provided by μOCT, analysis of the imaging results allows the neutrophil migration volume to be quantitatively determined permitting accurate estimates of the number of neutrophils migrating or having migrated at any given time. This was accomplished by performing simple thresholding and voxel counting (Fig. 4C–F), and agreement with MPO-derived results validated μOCT measurements. These results further illustrate that longitudinal imaging is a significant advantage, allowing μOCT to be more efficient in terms of samples consumed and frequency of sampling, while eliminating inter-sample variability as a confounding variable to time. The overall number of neutrophils in the single-well longitudinal analysis by μOCT was lower than detected by MPO activity analysis (Fig. 4C–F). This magnitude difference, however, is likely due the fact that migration assays that were analyzed by MPO activity were conducted in a 5% CO_2_ incubator whereas this environment was not available during μOCT analysis. Overall these data suggest that quantification by μOCT image analysis is accurate in comparison to existing methods and offers the advantage of single well analysis over time.

The utility of both the qualitative and quantitative μOCT observations is most clearly demonstrated where neutrophil trans-epithelial migration is experimentally disrupted using existing knowledge of the molecular mechanisms that underlie this biological process to evaluate the impact on neutrophil and epithelial cell behavior. Figures 5, 6 show the effect on migration when neutrophils were pre-treated with anti-CD18 antibodies that are known to interfere with fMLP directed neutrophil migration across T84 monolayers^16^,^26^. CD18, or beta-2 integrin, associates with CD11b forming the heterodimeric Mac-1 complex that is expressed on the surface of neutrophils. Mutation of CD18 is known to cause Leukocyte Adhesion Deficiency I^21^. We selected a concentration of anti-CD18 antibody that reduces but does not fully prevent transmigration allowing us to observe the nature of defect rather than fully incapacitating neutrophil trans-epithelial migration (Supplementary Figure 3).

Our μOCT results (Figs 5 and 6, Video 7) demonstrated greater epithelial adhesion and clustering of neutrophils pretreated with anti-CD18, both with qualitative observations of migration behavior and with quantitative analysis. By applying our threshold/voxel counting analysis, we could generate quantitative data for comparing neutrophil migrations in the presence and absence of anti-CD18 treatment. Both visual and numerical evidence showed that anti-CD18 treated neutrophils exhibited slightly decreased migration and significantly increased adhesion. Ideally the biological significance of this event would be better demonstrated using neutrophils isolated from patients with Leukocyte Adhesion Deficiency I^16^,^26^ as we cannot rule out possible effects associated with antibody crosslinking, however, our results clearly illustrate the capacity of μOCT to reveal aberrant cellular mechanisms previously inaccessible by traditional imaging techniques.

The quantitative data shown in Fig. 6A and B demonstrate the benefits of longitudinal μOCT imaging. Noise between samples was low since the same regions of the same samples were measured, and non-destructive acquisitions at 10 minute intervals did not require a cumbersome number of samples for multiple MPO assays. Perhaps a more powerful feature of μOCT-based assays is the ability to spatially localize the neutrophils post-migration. Attached and detached neutrophils could be discriminated and precise migration distances could be measured, which enabled the most illuminating results from this experiment; the differences in adhesion ratio and migration distance were statistically significant, even while total migration volumes were not. The true complexity of the abnormal behavior exhibited by anti-CD18 treated neutrophils was revealed only by μOCT and was not captured by simple myeloperoxidase quantification assays.

The μOCT advantages of longitudinal experimentation and spatial localization greatly enrich basic neutrophil-counting assays of migration. Both the adhesion ratio assay and migration distance assay may be useful for interrogating defects and anomalies in neutrophil adhesion. The observation that neutrophils tend to cluster during migration suggestions that neutrophil-neutrophil interaction is an important factor in the migration process. This possibility is further raised by evidence that anti-CD18 treated neutrophils adhered and clustered more durably after migration than control neutrophils. μOCT provides the unique means to observe this phenotype, and to determine its link to the decrease in migration volume. Furthermore, as more analyses are conducted applying distinct reagents that interfere with neutrophil trans-epithelial migration, additional observations will likely lead to the design of additional metrics that will be informative towards understanding neutrophil behavior.

## Scientific and Medical Significance

During infection or pathological mucosal inflammation emanating from auto-inflammatory conditions, neutrophils are known to traffic to the affected mucosa and breach the epithelial barrier by migrating across the protective epithelial layer. This process is thought to have important beneficial consequences as well as the potential to contribute to pathology and mucosal tissue damage creating a therapeutic paradox^16^. Neutrophils entering the mucosa are critical for containing pathogens and evidence is emerging that they may contribute to resolution of epithelial breaches. However, neutrophils release noxious proteases and reactive oxygen species that promote tissue damage and the migration across the barrier in and of itself can diminish barrier integrity. Inflammatory bowel disease is a condition thought to involve overzealous neutrophil infiltration with unclear benefit, however, therapeutic strategies that universally eliminate neutrophil recruitment to and across the mucosa to alleviate tissue damage may have unintended consequences. Clearly a more precise understanding of the molecular and cellular mechanisms that drive neutrophil penetration of the mucosal barrier would inform therapeutic attempts that modulate this process to a beneficial end. The novel μOCT imaging methodology described herein has enormous potential to quantitatively study the dynamics of neutrophilic breach of mucosal barriers by illuminating cellular mechanisms that can be interrogated with reagents that perturb known or hypothesized molecular mechanisms providing an integrated understanding of this important inflammatory event.

## Conclusion

Live imaging of neutrophil migration across epithelial barriers without need for sample manipulation enabled by μOCT technology represents a unique method for exploring an important inflammatory event that occurs in a variety of diseases. Observational and quantitative metrics described here provide a foundation for a completely novel means to explore live unadulterated co-culture models and reveal dynamic interactions that occur between cells in a complex biological system.

## Additional Information

**How to cite this article**: Chu, K. K. *et al*. Illuminating dynamic neutrophil trans-epithelial migration with micro-optical coherence tomography. *Sci. Rep.* **7**, 45789; doi: 10.1038/srep45789 (2017).

**Publisher's note:** Springer Nature remains neutral with regard to jurisdictional claims in published maps and institutional affiliations.

## Supplementary Material

Supplementary Information

Supplementary Video 1

Supplementary Video 2

Supplementary Video 3

Supplementary Video 4

Supplementary Video 5

Supplementary Video 6

Supplementary Video 7
