# Supplementary material for: Illuminating dynamic neutrophil trans-epithelial migration with micro-optical coherence tomography
Source: Sci Rep. 2017 Apr 3;7:45789. doi: 10.1038/srep45789 (PMC5377939; doi:10.1038/srep45789)
Supplement: Supplementary Information [file srep45789-s1.pdf]

# Supplementary Figures for "Illuminating dynamic neutrophil trans-epithelial migration with micro-optical coherence tomography"

Kengyeh K. Chu, PhD[1,3], Mark E. Kusek, MD[2,4], Linbo Liu, PhD[1,3], Avira Som[3,4], Lael M. Yonker, MD[2,4], Huimin Leung, PhD[1,3], Dongyao Cui[1,3], Jinhyeob Ryu[3], Alexander D. Eaton[4], Guillermo J. Tearney, MD, PhD[1,3,5*], Bryan P. Hurley, PhD[2,4*]

[1]Department of Dermatology, [2]Department of Pediatrics, Harvard Medical School, Boston, MA, USA
[3]Wellman Center for Photomedicine, [4]Mucosal Immunology and Biology Research Center,
[5]Department of Pathology, Massachusetts General Hospital, Boston, MA, USA

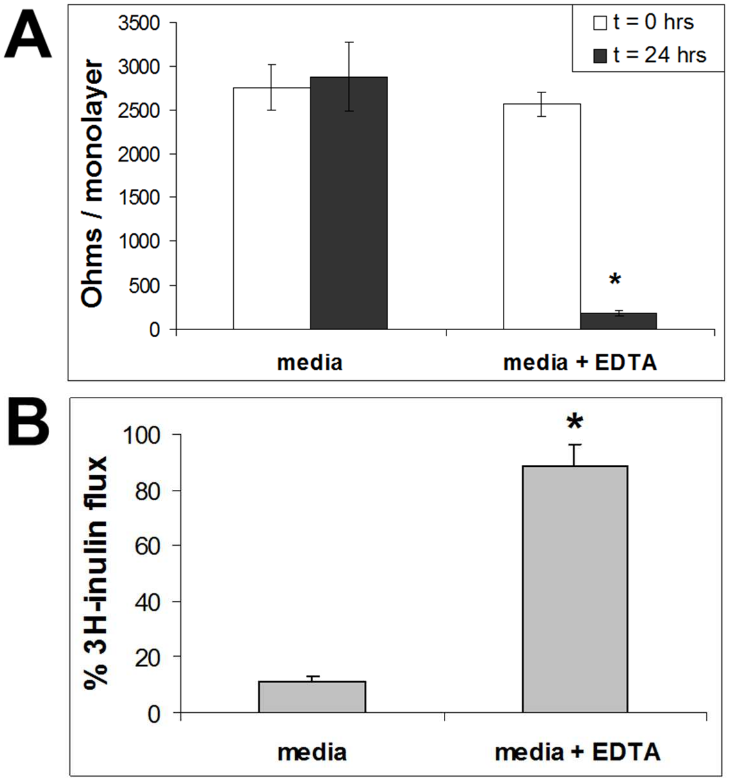


*Supplementary Figure 1: (A) Trans-epithelial electrical resistance (TEER) of the T84 epithelial barrier is measured at time 0 and 24 hrs following treatment in media with or without 5 mM EDTA. (B) The amount of 3H-inulin (3 kDA) that moves across the T84 barrier over a 24-hr period in the presence of media alone or media + 5 mM EDTA. If complete 3H-inulin equilibration is achieved suggesting an absence of barrier function a theoretical value of 85.7% would be expected. (*) denotes a significant difference from media control.*

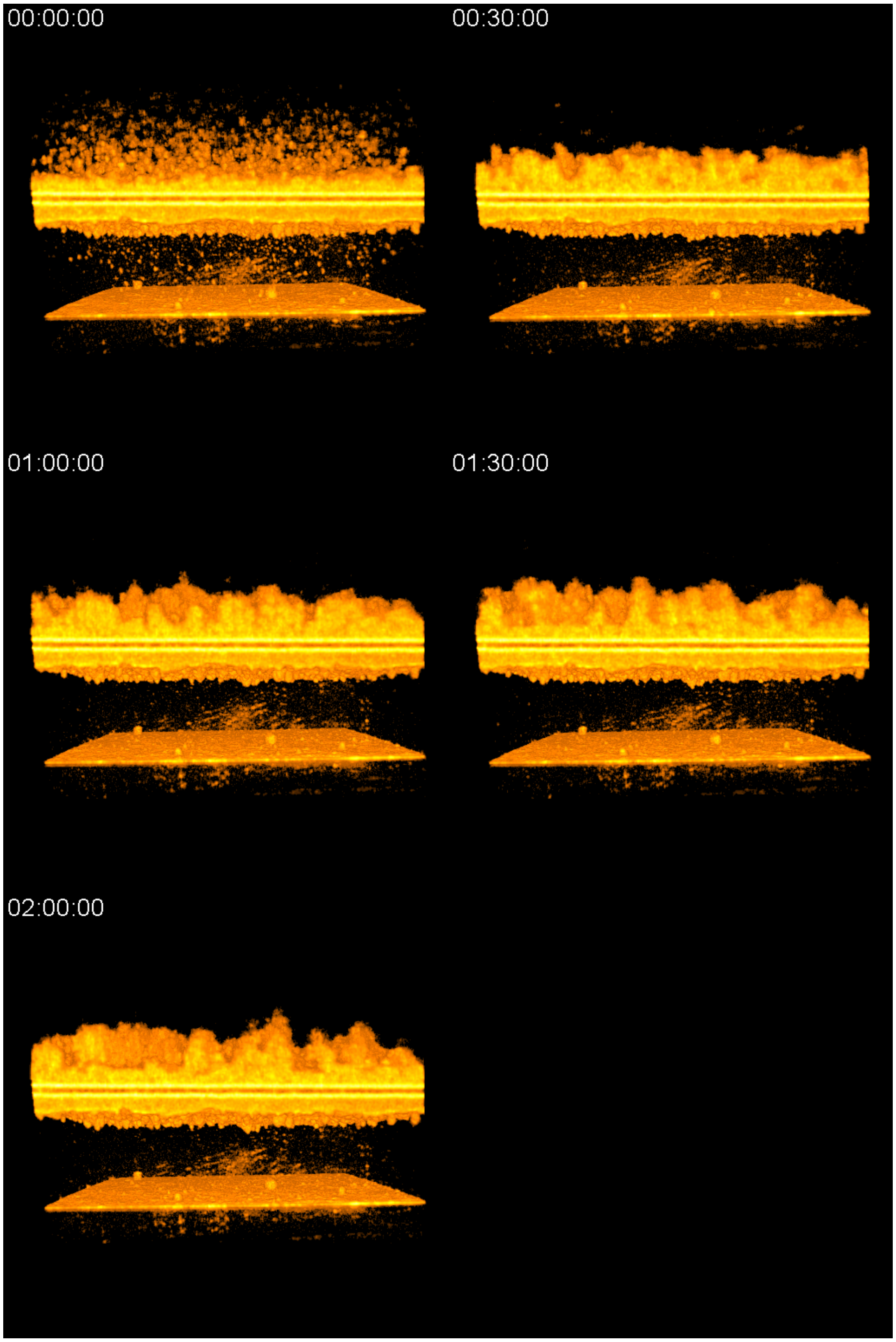


*Supplementary Figure 2: 3D renderings from µOCT 2-hour time lapse of neutrophil migration through T84 epithelium driven by HBSS+ negative control (room temperature).*

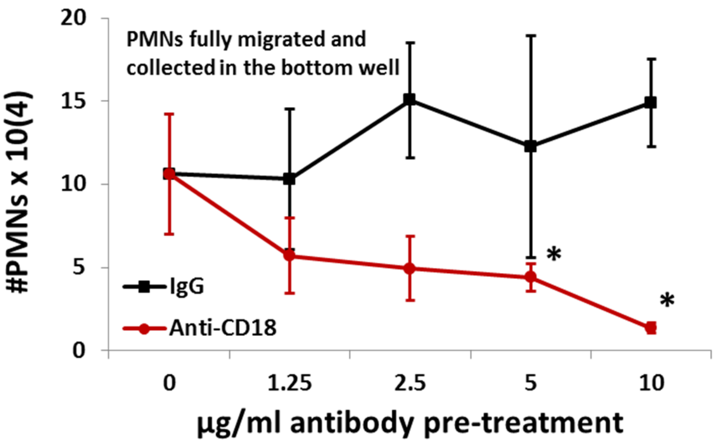


*Supplementary Figure 3: PMN migration dependence on antibody pretreatment concentration, MPO assay. Anti-CD18 induced dose-dependent migration suppression, while isotype control (IgG) did not. The symbol (*) indicates a statistically significant decrease from migration in the absence of antibody (0 µg/ml antibody pre-treatment)*

## Video captions

*Video 1: Scanned µOCT cross section across T84.*

*Video 2: Progression of µOCT XZ cross-sectional images of T84 epithelium, scanned in the Y direction (perpendicular to plane of imaging). Left: Prior to EDTA application. Right: 2 hours after EDTA application.*

*Video 3: Time lapse of µOCT XZ cross-sectional images of T84 epithelium during exposure to EDTA beginning at time 0. During the 2-hour exposure period, the apical surface appears to reduce in brightness and increase in roughness.*

*Video 4: µOCT cross-sectional XZ movie of neutrophils migrating across T84 epithelium in response to fMLP application to apical medium. Neutrophils settle in the first few minutes due to gravity, and begin to penetrate the epithelium in discrete clusters. Even after the neutrophils cross the epithelium, they appear to remain adhered to each other. As migration continues, neutrophils from some larger clusters detach. Scale bar: 50 µm. Time labels are hh:mm:ss.*

*Video 5: Time lapse 3D µOCT renderings of neutrophil migration through T84 epithelium driven by fMLP chemoattractant (room temperature). Neutrophils added to the basolateral side of the epithelial barrier at time 0 penetrate the epithelium in discrete clusters over a 2 hour imaging period. A fraction of the neutrophils detach from the clusters and fall to the glass surface. Left: 3D perspective view along a lateral aspect. Right: 3D bottom-up view of the apical epithelium. See Figure 4 for labeled features.*

*Video 6: µOCT cross-sectional XZ movie of neutrophils treated with anti-CD18 antibodies migrating across T84 epithelium in response to fMLP application to apical medium. Compared to untreated neutrophils, the anti-CD18 PMNs appear to remain attached to the epithelium in greater proportion (compare to Video 4). Scale bar: 50 µm. Time labels are hh:mm:ss.*

*Video 7: Time lapse 3D µOCT renderings of neutrophil migration, treated by anti-CD18 antibodies (top) and IgG1 isotype control (bottom) through T84 epithelium driven by apical fMLP. IgG1 treated neutrophil migration appears qualitatively similar in volume and character to untreated neutrophils, while anti-CD18 treated neutrophils appear to remain attached to the epithelium in large hanging clusters. Left: 3D perspective view along a lateral aspect. Right: 3D bottom-up view of the apical epithelium.*
